# An Automated Virtual Reality Training System for Teacher-Student Interaction: A Randomized Controlled Trial

**DOI:** 10.2196/41097

**Published:** 2022-12-08

**Authors:** Seth King, Joseph Boyer, Tyler Bell, Anne Estapa

**Affiliations:** 1 Department of Teaching and Learning College of Education University of Iowa Iowa City, IA United States; 2 Department of Electrical and Computer Engineering College of Engineering University of Iowa Iowa City, IA United States

**Keywords:** virtual reality, artificial intelligence, behavioral skills training, education, professional development, staff training, mathematics

## Abstract

**Background:**

Shortages in qualified supervision and other resources prevent education personnel from rehearsing effective practices. Interactive simulations, although increasingly used in education, frequently require instructor management. Automated simulations rarely engage trainees in skills related to practice (eg, speech).

**Objective:**

We evaluated the capability of delivering behavioral skills training through an automated virtual reality (VR) simulation using artificial intelligence to improve the implementation of a nondirective mathematical questioning strategy.

**Methods:**

We recruited and randomly assigned 30 college-aged participants to equivalent treatment (ie, lecture, modeling, and VR; 15/30, 50%) and control groups (ie, lecture and modeling only; 15/30, 50%). The participants were blind to treatment conditions. Sessions and assessments were conducted face to face and involved the use of VR for assessment regardless of the condition. Lessons concerned the use of a nondirective mathematical questioning strategy in instances where a simulated student provided correct or incorrect answers to word problems. The measures included observed and automated assessments of participant performance and subjective assessments of participant confidence. The participants completed the pretest, posttest, and maintenance probes each week over the course of 3 weeks.

**Results:**

A mixed ANOVA revealed significant main effects of time (*F*_2,27_=124.154; *P<*.001; η*_p_*^2^=0.816) and treatment (*F*_1,28_=19.281; *P<*.001; η*_p_*^2^=0.408) as well as an interaction effect (*F*_2,28_=8.429; *P<*.001; η*_p_*^2^=0.231) for the average percentage of steps in the questioning procedure. Posttest scores for the intervention group (mean 88%, SD 22.62%) exceeded those of the control group (mean 63.33%, SD 22.64%), with *t*_28_=3.653, *P*<.001, and Cohen *d*=1.334. Maintenance scores indicated a positive effect of the intervention (mean 83.33%, SD 24.40%) relative to the control (mean 54.67%, SD 15.98%), *t*_28_=3.807, *P*<.001, Cohen *d*=1.39. A Mann-Whitney *U* test indicated that the treatment groups’ self-ratings of confidence (mean 2.41, SD 0.51) were higher than those of the control group (mean 2.04, SD 0.52), *U*=64, *P*=.04, *r*=0.137.

**Conclusions:**

The results demonstrate the potential of artificial intelligence-augmented VR to deliver effective, evidence-based training with limited instructor management. Additional work is needed to demonstrate the cascading effect of training on authentic practice and to encompass a wider range of skills.

## Introduction

### Background

High-quality professional development contributes to the effectiveness of education personnel [1] and success of their students [2]. However, the training education professionals receive before entering the field often consists of lectures [3] with few opportunities to practice skills or receive performance feedback—key aspects of effective professional development [4-6]. Training ideally provides multiple opportunities for practice across a range of unpredictable situations under the supervision of a competent observer [7]. The prevalence of less effective approaches to professional development stems from a shortage of qualified supervisors and suitable practicum placements in many areas [6,8]. The difficulty in providing effective training for education personnel has perpetuated the use of ineffective practices in education [9].

Head-mounted virtual reality (VR) using an array of visual, auditory, and tactile interfaces that adjusts the display based on user sensorimotor inputs to fully immerse participants within a simulation [10] is increasingly associated with improved learner outcomes, increased engagement, and the ability to repeatedly practice skills [11,12]. The immersion permitted by VR potentially allows educators to engage in behaviors and interact with stimuli closely aligned with actual practice, which can potentially improve the administration of instruction and increase teacher confidence [13]. The maximum immersion permitted through VR may not be necessary in all scenarios (eg, conversation); however, VR allows for a realistic representation of nonverbal communication that accompanies speech as well as any potential physical interactions. In addition, *removing* components from a fully immersive simulation to place core content on platforms such as desktops is technically easier than *adding* critical immersive elements to simulations developed on less-sophisticated devices [14]. VR applications can be adapted to preserve their core functionality across a range of devices, thus making development on the platform potentially conducive to dissemination.

The technology associated with VR has become more affordable [15]; however, it is most frequently used as a training tool in medicine [16]. The results of VR simulations tested in the educational context are mixed, with early reports of limited effectiveness and induced illness [15] being contradicted only by recent research with more positive findings [17]. VR simulations specific to teacher education typically require trainees to observe events depicted in 3D space (eg, bullying behavior and self-injury) rather than interact [13,18]. VR simulations targeting human interaction are typically controlled directly by expert trainers; at a minimum, human observers must administer assessments [13]. Experiments involving the performance of complex procedures (eg, functional communication training [19]), although they include scripts to simulate the behavior of student avatars, rely on researchers to assess trainees.

Although absent from studies of teacher training, artificial intelligence (AI)—or software capable of independently acquiring, processing, and acting upon information [20]—is emerging as a force in education through the growing implementation of chatbots, automated assessment, facial recognition, and other functions to support teaching and learning [21]. AI relies on machine learning (ie, natural language processing) in which computers are trained to classify new stimuli following exposure to previous data sets (ie, training data) and statistical models to make predictions based on new information [22]. Intelligent tutoring systems, which provide individualized instruction based on the responses and characteristics of learners, encompass many AI functions relevant to education, including learner assessment, content generation, and providing feedback [23]. An increasing number of programs targeting specific academic skills in K-12 and higher education have recently emerged [22], such as the IBM Watson Tutor, a dialogue-based tutor that uses natural language processing to interpret learners’ comments and provide appropriate feedback [24]. However, studies integrating VR and AI are currently limited [22]. Existing applications incorporating AI, such as Lamb and Etopio’s classroom management scenarios [25], allow for participant interaction with student avatars but do not assess the implementation of discrete instructional practices.

A recent experiment [14] demonstrated the effectiveness of an automated VR simulation capable of administering instructional procedures and assessing learner performance on the use of a mathematical questioning procedure. Evidence suggests that nondirective mathematical questioning, in which students’ thought processes are elicited before confirming whether an answer is correct, can improve student outcomes [26]. Effective questions require students to assess, explain, and justify their answers [27]. This process facilitates instructional decisions, especially when teachers cannot observe the problem-solving process or the correct answer may be derived through an inefficient or inappropriate approach [28]. Interaction-focused techniques such as mathematical questioning, which involves the assessment of speech rather than simple movements or button presses, differ from the content generally addressed in VR training simulations.

The training developed by King et al [14] consisted of video-recorded lectures and simulations capable of providing automatic assessment, textual prompting, and feedback through the incorporation of VR and AI (eg, speech classification and speech-to-text). As in the research conducted by Clay et al [19], the components of the intervention were arranged in accordance with behavioral skills training (BST), an evidence-based approach to personnel preparation encompassing a range of instructional components, including didactic instruction, modeling, rehearsal, and feedback [29,30]. Textual onscreen prompts, systematic prompting, and video models were incorporated based on evidence of their effectiveness in the literature [31-33]. The use of a single-case design [34-36] permitted improvements in simulation functionality over the course of the experiment, resulting in automated assessments with a high degree of agreement with direct observation (>96%) and large changes in the percentage of steps in the procedure exhibited by the two participating trainees after 3 consecutive days of training (Tau_bc_=0.80 [37]).

In contrast to many approaches to VR, which are not constructed in accordance with a specific learning theory [38], the simulation developed by King et al [14] was predicated on behavioral theories of learning and instruction [39] that aim to encourage appropriate responses in the presence of specific antecedents (ie, discriminative stimuli), for example, praising a student for correctly answering a math problem. The ability of an antecedent to evoke the correct response can be increased and sustained through the introduction and gradual fading of prompts. Instructors may also administer consequences designed to increase correct responses, which can include providing stimuli of value to the learner (ie, positive reinforcement) or allowing the learner to avoid unpleasant stimuli (ie, negative reinforcement) [39]. Prompts and consequences are most effective when provided immediately. In King et al [14], the responses generated by simulated students represented antecedents associated with the correct steps of a procedure. The participants received textual prompts before they had an opportunity to respond. In addition, the simulation provided correctives immediately following incorrect responses and required the participants to provide a correct response. The avoidance of corrective procedures upon the use of correct responses in subsequent sessions and feedback regarding correct answers following each session provided negative and positive reinforcement, respectively. Notwithstanding this theoretical basis and the positive findings associated with the simulation, the small sample size and iterative development that occurred over the course of the experiment represent clear limitations.

### Purpose

Given the scarce resources available for training, a simulation capable of independently providing assessment and instruction related to student-teacher interaction has the potential to benefit education personnel as well as their students and result in the wider dissemination of professional development. In light of the limitations of earlier work in this area [14], this study assessed the ability of a feature-locked, AI-enhanced VR training application to independently impart the steps of a mathematical questioning strategy using a randomized controlled design. The guiding questions included the following: (1) Compared with individuals who did not receive training in VR, does the simulation improve the participants’ acquisition of steps in a mathematical questioning strategy? (2) To what extent does group performance differ during maintenance (ie, extended absence of instruction) and generalization (ie, untaught items) probes? (3) Does the VR simulation increase the participants’ perceived confidence in the use of the procedure, relative to the control group? (4) To what extent do the observed measures of trainee performance correspond with the automated measures?

## Methods

### Ethics Approval

The university institutional review board at the University of Iowa approved all procedures and consent forms before recruitment (202112205).

### Participants and Setting

Recruitment began in January 2022. The study was conducted throughout March and April 2022. Eligible participants were current and former graduate and undergraduate students affiliated with the University of Iowa. Potential participants were (1) aged >18 years; (2) free of seizure disorders, epilepsy, or other health conditions potentially exacerbated by VR; and (3) able to use voice-activated assistants such as Alexa without accommodations. In addition, we excluded participants who were likely to be familiar with the subject material (ie, participants with employment experience in an educational setting or participants with records of completing courses in mathematics education) to ensure sufficient sensitivity to the intervention. A US $15 gift card was offered as an incentive for participation. For recruitment, the second author described the study to students in cooperating classrooms; the participants were also encouraged to share information regarding the study with potentially interested peers. In total, 30 individuals agreed to participate in this study. We assigned the participants to the experimental groups using a stratified randomization [40] procedure based on observed mastery probes (OMPs) during the baseline phase. No attrition occurred over the course of the study. A survey of participant characteristics revealed no significant differences in familiarity with VR before the experiment. Additional demographic characteristics of the participants are shown in Table 1.

Sessions and assessments were administered face to face in a small room with computer and internet access. Each participant’s sessions occurred once per week for 3 consecutive weeks. Scheduling ensured that approximately 7 days elapsed between the assessment sessions, which otherwise occurred at times acceptable to the participants. The participants were advised to terminate the sessions at the first sign of discomfort; however, all the sessions were completed without any incident. A master’s-level student in computer engineering (ie, the session administrator) conducted all the sessions with the participants individually.

**Table 1:** Participant demographics.

Participant		Total (N=30)	Control (n=15)	VR^a^ (n=15)
Age (years), mean (SD); range		22.13 (1.5); 19-26	21.87 (1.81); 19-26	22.4 (1.12); 21-25
**Sex^b^, n (%)**				
	Male	16 (53)	8 (53)	8 (53)
	Female	14 (47)	7 (47)	7 (47)
**Academic program, n (%)**				
	Business or finance	8 (27)	4 (27)	4 (27)
	Engineering	13 (43)	6 (40)	7 (47)
	Other	9 (30)	5 (33)	4 (27)
**Educational status, n (%)**				
	Undergraduate	15 (50)	9 (60)	6 (40)
	Masters	6 (20)	2 (13)	4 (27)
	Doctoral	2 (7)	2 (13)	0 (0)
	Other	7 (23)	2 (13)	5 (33)
VR experience^c^, mean (SD); range		4.47 (1.74); 1-6	4.47 (1.73); 1-6	4.47 (1.81); 1-6

^a^VR: virtual reality.

^b^None of the participants identified as being nonbinary.

^c^VR experience was determined using a 6-item Likert-type scale ranging from 1 (no experience) to 6 (much experience).

### Materials

A commercially available Windows (Microsoft Corp) desktop computer facilitated video playback. We used the Oculus Quest 2 (Facebook Reality Labs; US $300) VR headset and its 2 controllers for all the instructional simulations. The Quest device has an integrated microphone and speakers and tracks motion without external sensors.

### Dependent Variables

#### Overview

This study examined 6 distinct dependent variables. The observed percentage of lesson steps completed correctly (ie, OMP) represented the principal outcome. To evaluate the simulation’s assessment capabilities, we compared the results of the OMPs with those of a virtual mastery probe (VMP) assessing the same skills. We obtained additional information regarding the perceptions of the participants from the SKIL Survey [41].

#### OMP Assessment

For the OMPs, the session administrator collected information regarding the percentage of steps in the questioning procedure that the participants completed while interacting with the student avatar in the VR simulation. We calculated the results in terms of the total number of steps performed correctly divided by the total number of steps in procedures taught in lessons 1 and 2 combined (ie, 10 steps). The OMPs were created in relation to the content taught during training (ie, *acquisition* probes) to assess the participants across the baseline, posttest, and maintenance sessions as well as untaught content to assess the generalization of the procedure in posttest and maintenance sessions (ie, *generalization* probes). The steps in the acquisition probe are shown in Table 2 (refer to “teacher steps”). We scored the OMPs in accordance with the single-opportunity method, in which the probe ended as soon as the participant exhibited an incorrect response [42] because of (1) concerns regarding time commitment and (2) the chance of skill acquisition in the absence of instruction. Research suggests that single-opportunity method probes of chained tasks contribute relatively little bias [42]. The participants did not receive feedback following the completion of the OMPs.

**Table 2:** Steps in lessons 1 and 2 for teachers and simulated student.

Step		Description	Example and variations
**Lesson 1: responding to a correct answer**			
	T1^a^: read the problem	The teacher reads the problem clearly and without errors	“You have 5 fishbowls with 4 fish in each bowl. How many fish are there total?”
	S1^b^: brief student correct answer	The student provides the correct answer without additional detail	“There are 20 fish.” “The answer is 20.”^c^
	T2: unpack strategy request (correct)	After the student provides the correct answer, the teacher asks the student to explain	“How did you solve this problem?” “Why is that the answer?”
	S2: student unpacks correct strategy	The student describes the appropriate method they used for answer	“I multiplied 5 times 4.” “I took 5 and 4 and multiplied.”
	T3: Praise	The teacher praises the student’s effort	“Good job.” “Nice job, buddy.”
**Lesson 2: responding to an incorrect answer**			
	T1: read the problem	—^d^	—
	S1: brief student incorrect answer	The student provides an incorrect answer without additional detail	“I don’t know. Nine fish?” “It’s nine fish I think.”
	T2: unpack strategy request (incorrect)	After the student provides an incorrect answer, the teacher asks the student to explain	—
	S2: student unpacks incorrect strategy	The student describes the inappropriate method they used for answer	“I added 5 plus 4.” “I used addition.”
	T3a: underscore task feature (strategy)	The teacher asks why the student used a specific incorrect strategy	“What in the problem made you add?” “Tell me why you used addition.”^e^
	S3: strategy explication	The student describes why they used an incorrect strategy	“Well, you said there were 5 fishbowls and 4 fish.” “I didn’t know what to do, so I added.”
	T3b: underscore task feature (problem)	The teacher prompts the student to re-examine the problem	“What is the problem asking you to do?”
	S4: feature identification	The student proposes a new approach based on the problem features	“I see. I need to count the fish in all of the bowls.” “I need to add five ‘4s’ together.”
	T4: teacher grouping request	The teacher asks the student to attempt the problem again	“What would your answer be now?” “Can you try solving again?”
	S5: brief student correct answer	The student provides the correct answer without additional detail	“You would have 20 fish then.” “The answer is 20.”
	T5: unpack strategy request (correct)	—	—
	S6: student unpacks correct strategy	—	—
	T6: praise	—	—

^a^T: teacher.

^b^S: student.

^c^To prevent rote responding, the students provided varied responses for each step. Some examples are not exhaustive.

^d^The content is identical to previous version of the step.

^e^Variations for teachers refer to potentially correct examples. Examples are not exhaustive.

#### SKIL Survey

We assessed the respondents’ stated understanding of questioning using an adapted version of the SKIL Survey [41]. The survey consisted of concepts rated across three scales: (1) *knowledge* of the content, (2) *confidence* in the use of the techniques, and (3) perceived *usefulness*. The respondents rated the items using a 4-point Likert-type scale ranging from 0 (eg, *no knowledge*) to 3 (eg, *substantial knowledge*). We presented a small sample of eight assessment items because of the narrow focus of the training. Surveys featuring a reduced number of items obtained acceptable internal consistency in previous studies, with a Cronbach α for knowledge of .907, confidence of .882, and usefulness of .915 [13]. We delivered instructions regarding the purpose of the assessment before each administration. The participants completed the SKIL Survey during the baseline and maintenance sessions.

#### VMP Assessment

The VMP and OMPs were administered concurrently to determine the correspondence between the simulation-administered assessments and direct observations conducted by a human. The VMP assessed the exact same steps in the procedure as the acquisition and generalization OMPs via the speech classifier embedded in the simulation, which (1) recorded textual output corresponding to a participant’s spoken response, (2) determined whether the text’s classification matched the classification of correct responses for each step, and (3) calculated the percentage of correct steps completed by the participant.

### Design

We analyzed the effectiveness of the intervention using a single-blind, independent measures pretest-posttest design. The participants were placed in an intervention condition (ie, lecture, model, and VR practice) or a control condition (lecture and model only) using stratified random assignment [40] based on baseline OMP scores. Randomization was achieved using Microsoft Excel. The identities of the participants were concealed from the researchers during the randomization process.

### Simulation

**Figure 1:**
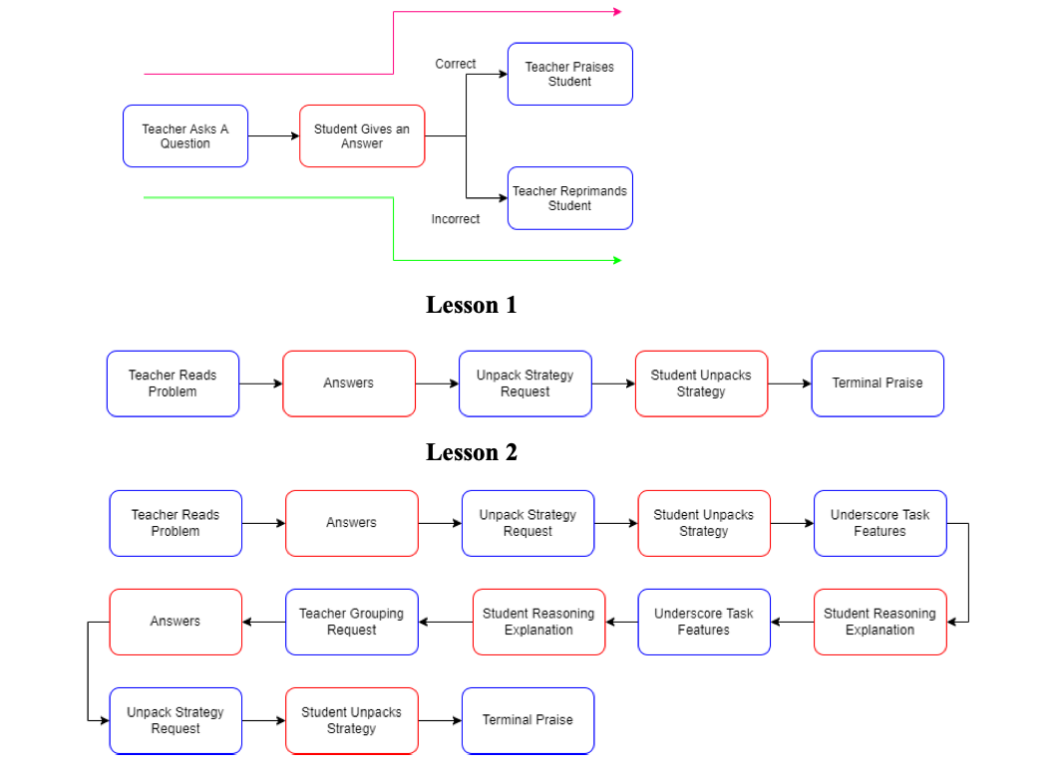
Flowcharts depicting example of appropriate sequence of events in a scenario (top), steps in lesson 1 (middle), and steps in lesson 2 (bottom).

The VR simulation featured in this study was developed over the course of 2 years by an interdisciplinary team consisting of faculty in behavior analysis, math education, and computer engineering. A task analysis [39] of mathematical questioning was conducted to identify the teacher and student actions emitted during applications of mathematical questioning. In a departure from the typical task analysis procedure, we created different lessons based on likely student responses. The initial analysis included many possible variations accounting for student actions such as nonresponses. This became the basis for the skills evaluated in this study: (1) responding to a correct answer and (2) responding to an incorrect answer (ie, lessons 1 and 2). An example of the flowcharts resulting from this process that illustrate the possible sequences of events in a scenario, as well as sequences in lessons 1 and 2, are shown in Figure 1. Table 2 describes the specific steps in each lesson.

**Figure 2:**
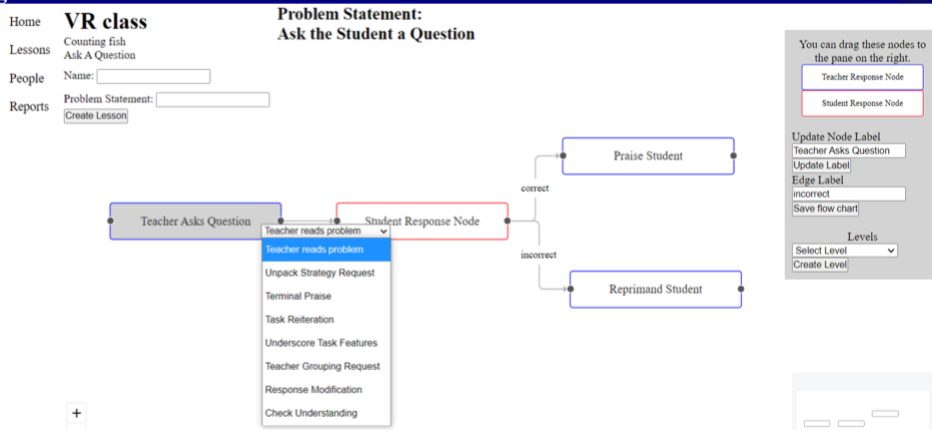
Web application page depicting tools used to design flowcharts and novel lessons. Lesson creation provides a drag and drop interface to allow the creation and connection of nodes for a flowchart. In addition, classifications can be assigned to each node. Once the flowchart is completed, the lesson creation page also allows the user to create individual lessons needed to run a simulation. VR: virtual reality.

**Figure 3:**
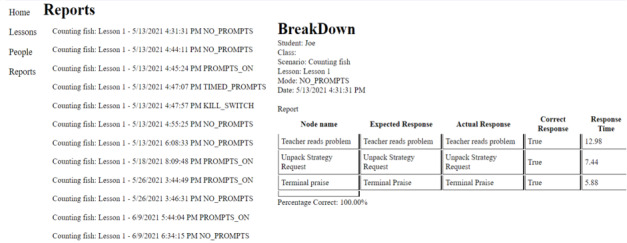
Reports page depicting simulation feedback. The report includes all the information recorded by the simulation feedback. Having reports accessible allows the instructors to create personalized analyses for each trainee and create a profile to determine how effectively the trainee understands the presented material.

**Figure 4:**
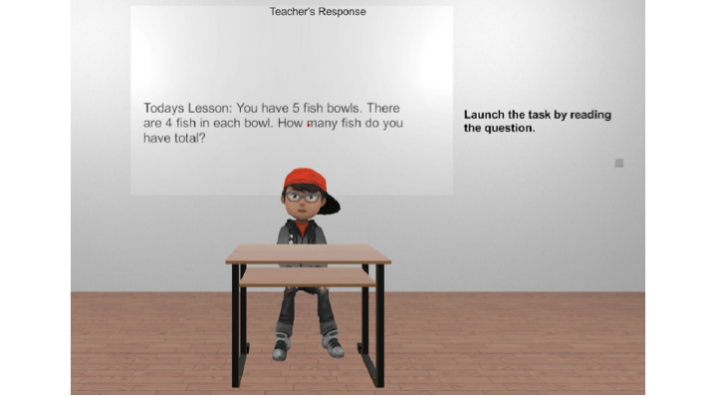
Depiction of basic virtual scenario and textual user prompt.

The simulation corresponding to the mathematical questioning procedure consisted of multiple components. A custom React [43] web application permitted the generation of simulation content (eg, steps in a procedure), which was stored in a database for retrieval by the simulation. Figure 2 depicts the web application used to generate flowcharts, allowing for different lessons based on the anticipated student responses. The application further allowed for the generation of reports regarding the performance of the participants (Figure 3). The simulation, developed in Unity (Unity Technologies), allows trainees to interact with a virtual student in a simulated classroom environment. All VR assessments and instructional sessions across experimental conditions began with the participant verbally presenting the student avatar with a math problem involving multiplication before deviating into different pathways based on the initial student response. A depiction of the start of a typical simulation and user prompt is shown in Figure 4. We trained the speech classification AI to recognize the topographical variations of potential participant statements. The key technical aspects of this work include (1) the ability of the virtual student to speak to the participant, (2) the ability of the participant to vocally respond to the virtual student, and (3) the ability of the simulation to classify the participant’s responses as correct or incorrect.

Speech from the student avatar was accomplished using IBM Watson’s [44] Text-to-Speech, which converted text strings corresponding to predetermined student responses into audio data. To promote the extent to which trainees responded correctly to distinct student statements that should nonetheless evoke a similar step in the procedure (eg, incorrect response; nonresponse) [45], student avatar responses at each step were drawn from functionally identical yet topographically dissimilar text strings. Examples of the student statements are listed in Table 2.

Assessment of the trainees’ responses was accomplished using IBM’s Speech-to-Text, which converted the trainees’ statements into a text string, and the Google Natural Language AI service [46], which determined whether a trainee’s transcribed statement matched the targeted response for a specific step of the procedure. The text classifier was trained using phrases corresponding to each step of the simulation (Table 2). After training, the classifier could be used to identify novel text strings that did not perfectly match the training phrases. This allowed the simulation to accommodate variability in the trainees’ responses. The classifier would provide a confidence value between 0.00 and 1.00, specifying the degree to which the provided text corresponded to each step of the procedure. Higher values reflected a greater degree of confidence in a statement’s correspondence to the phrases included in the training. We established a classifier threshold (eg, 0.75) to determine how closely the participants’ responses needed to match the expected response at each step. If the classification confidence exceeded the threshold, the system identified the participants’ responses as correct.

When combined with recorded lectures describing the rationale for a procedure and a model of the procedure’s delivery, the use of the VR simulation comprised a computer-mediated form of BST. Resources associated with the appropriate delivery of BST have often prevented its use in practice [47]. Consequently, automating instructor-intensive portions of the practice may assist in disseminating effective training practices.

### Procedures

#### Baseline

During the baseline session, the participants completed a brief demographic probe and the SKIL Survey. To acclimate the participants to VR, the participants completed a brief custom tutorial introducing them to concepts such as the need to depress and hold the right trigger while speaking as well as the appearance of onscreen prompts. The participants then completed 2 OMPs related to lessons 1 (ie, student avatar responds correctly) and 2 (ie, student avatar responds incorrectly). For both lessons, the initial prompt presented on the Oculus display—a math problem based on content commonly featured in 3rd grade mathematics—was “You have 5 fish bowls. There are 4 fish in each bowl. How many fish do you have total?” Together, the 2 OMPs evaluated the participants’ ability to follow best practice over 10 teacher responses. The OMP for each lesson terminated immediately following an incorrect response. Although the system classified and scored each participant, the session administrator performed classifications manually to ensure an accurate assessment of the performance of the participants. Regardless of when the OMP was terminated, the simulation displayed “This concludes the session” at the conclusion of the probe. No further feedback was provided.

#### Training and Posttest Session

##### Overview

After the completion of the baseline assessments, the participants were randomly assigned to the control and experimental groups. Before assignment, we divided the entire sample into groups based on baseline OMP scores; members of these groups were then randomly assigned to the treatment conditions to ensure roughly equivalent baseline OMP scores for the control (mean 22, SD 4.140) and treatment groups (mean 22.67, SD 4.577). Sessions were conducted 1 week following baseline.

##### Control

The participants in the control condition watched a video-recorded lecture concerning the rationale and steps of the procedures for lessons 1 and 2. The lectures also included video models displaying educators using the procedures in practice with elementary-aged students. The lecture concluded with a description of the problem used in the OMP (ie, “You have 5 fishbowls…”) and a description of how the procedures would be applied to this specific problem. The duration of the lecture was approximately 12 minutes. Following the lecture, the participants completed the same OMP used during the baseline sessions. In addition, the participants completed a generalization OMP featuring a novel problem: “There are 4 buckets. There are 3 apples in each bucket. How many apples are there total?” The student avatar’s responses were adjusted to accommodate the new prompt. The generalization OMP terminated following the first incorrect response.

##### Intervention

The participants in the intervention group observed the same recorded lecture provided in the control condition. Thereafter, the participants received a series of supplemental VR trainings. For each lesson, the participants received 2 simulations of error-free prompting (EFPT), followed by 2 sessions of delayed prompting (DPT). EFPT simulations followed the general format of typical OMP; however, written examples of correct responses appeared on the screen immediately after each statement made by the student avatar. For DPT, prompts appeared on the screen following an incorrect response or nonresponse (ie, no response within 4 seconds). Immediately following each DPT, the simulation displayed the percentage of steps completed correctly and a description of the steps of each procedure missed (ie, performance feedback [48]). Classifications of participant performance, prompts, and feedback were all managed by the simulation without input from the session administrator. The combined duration of the supplemental VR trainings was approximately 10 minutes. The participants completed both the acquisition and generalization OMPs following training.

#### Maintenance

One week following the posttest sessions, the participants from both groups completed an additional acquisition OMP and generalization OMP in accordance with the procedures observed in the baseline and posttest sessions. The participants also completed an additional SKIL Survey as well as an assessment related to the acceptability of the training.

### Analysis

For OMPs, a 2-way mixed-design ANOVA was used. The analysis evaluated differences using a between-participants factor of treatment (control or intervention) and a within-participant factor of time (baseline, posttest, and maintenance sessions). Partial eta squared, η*_p_*^2^, was used to indicate the extent of group differences, with values of 0.02, 0.13, and 0.26 representing small, moderate, and large effects, respectively [49]. Statistically significant main effects, if observed, were followed by an analysis of simple effects using within- and independent samples *t* tests (2-tailed). Effect sizes were determined using Cohen *d*, with values of 0.8, 0.5, and 0.2 for large, medium, and small effects, respectively [50]. Sphericity, normality, and homogeneity were evaluated using Mauchly, Shapiro-Wilk, and Levene tests, respectively.

The differences between the SKIL Survey responses in the baseline and maintenance sessions were analyzed using the Mann-Whitney *U* test, a nonparametric alternative for comparing group differences [50]. Effect sizes were determined using Cohen *r*, with scores exceeding 0.5 representing a large effect, scores between 0.49 and 0.3 representing a moderate effect, and scores between 0.29 and 0.1 representing a small effect [51]. We initially examined differences in confidence, given that knowledge and usefulness ratings were likely to stem from didactic instruction (ie, information received by the participants rather than opportunities for practice), which did not differ between the 2 groups. In addition, the results of our previous research suggested that these 2 dimensions are insensitive to VR training [13]. However, we compared the findings across the knowledge and usefulness scales as an exploratory analysis.

Correspondence, defined as OMPs and VMP recording the same value (eg, correct or incorrect) for a participant’s response, was collected for each acquisition assessment, generalization assessment, and the VR simulations comprising the supplemental instruction. The calculations involved dividing the number of correspondences by the total number of responses and multiplying by 100.

Multiple comparison corrections were conducted for the 15 a priori statistical tests and 4 additional post hoc tests using the Benjamini-Hochberg procedure [52] with a false-discovery rate of 10%. [52] with a false-discovery rate of 10%. All raw *P* values, reported throughout, were significant following the Benjamini-Hochberg procedure unless indicated otherwise. All analyses were conducted using SPSS (IBM Corp).

### Interobserver Agreement

Interobserver agreement (IOA) was collected across all phases of the project. Specifically, a doctoral-level faculty member (ie, the secondary observer) with experience observing the completion of the mathematical questioning procedure [14] collected OMP data—including generalization probes—in 43% of the baseline sessions, 28% of the posttest sessions, and 20% of the maintenance sessions across treatment and control groups. The secondary observer’s results were compared with those of the session administrator. IOA was then calculated by dividing the number of agreements (ie, steps in the procedure in which observers recorded the same response) by the total number of steps in lessons 1 and 2 and multiplying the resultant number by 100%. The average IOA for the baseline and posttest sessions was 100% (SD 0%). The average IOA for the maintenance session was 100% for the experimental group and 93.33% (SD 9%; range 80%-100%) for the control group.

### Fidelity

We assessed the experimental protocols across all conditions using the checklists featured in our previous work [14]. The checklists indicated whether the session administrator delivered appropriate instructions, assessments, and simulation components. Fidelity was collected across numerous sessions in the baseline (43%), posttest (26%), and maintenance conditions (20%) and determined by calculating the percentage of steps for each session performed by the session administrator. The average baseline fidelity was 98.07% (SD 4.69%; range 87.50%-100%). In the posttest sessions, fidelity for the control and experimental groups was 100% and 95% (SD 5.77%; range 90%-100%), respectively. Fidelity across maintenance sessions was 100%.

### Acceptability

During the maintenance session, we used a consumer satisfaction survey featured in previous studies [13] to assess the acceptability of the simulation. The participants responded to statements concerning the project (ie, *The use of VR was acceptable to me; I had no difficulty using VR*) using a 6-item scale (1=*strongly disagree*; 6=*strongly agree*). The participants also answered a series of questions related to their experiences in the simulation.

## Results

### Overview

Descriptive statistics for baseline, posttest, and maintenance variables are listed in Table 3.

**Table 3:** Descriptive statistics for baseline, posttest, and maintenance variables across groups.

Variable			Total	Control	Intervention
**Pretest session**					
	OMP^a^ (percentage of correct responses), mean (SD); range		22.33 (4.30); 20-30	22 (4.14); 20-30	22.67 (4.58); 20-30
	**SKIL variables**				
		Knowledge, mean (SD); range	1.28 (0.47); 0.50-2.25	1.48 (0.49); 0.63-2.25	1.08 (0.37); 0.38-1.63
		Confidence, mean (SD); range	1.19 (0.61); 0-2.13	1.42 (0.60); 0.13-2.25	0.97 (0.54); 0-1.63
		Usefulness, mean (SD); range	1.73 (0.60); 0.38-2.75	1.83 (0.60); 0.38-2.5	1.63 (0.61); 0.5-2.75
**Posttest session**					
	**OMP**				
		Percentage of correct responses (% correct), mean (SD); range	75.67 (22.08); 40-100	63.33 (22.64); 50-100	88.00 (22.62); 40-100
		Generalization, percentage of correct responses, mean (SD); range	76.33 (24.70); 30-100	66.00 (22.62); 30-100	86.67 (22.89); 50-100
	**SKIL variables^b^**				
		Knowledge, mean (SD); range	2.36 (0.52); 1.25-3	2.27 (0.53); 1.25-2.88	2.45 (0.51); 1.5-3
		Confidence, mean (SD); range	2.23 (0.51); 1.38-2.88	2.04 (0.52); 1.25-2.75	2.41 (0.45); 1.5-2.88
		Usefulness, mean (SD); range	2.70 (0.32); 1.88-3	2.68 (0.35); 1.88-3	2.72 (0.30); 2-3
**Maintenance session**					
	**OMP**				
		Percentage of correct responses, mean (SD); range	69 (24.96); 30-100	54.67 (15.98); 30-100	83.33 (24.40); 50-100
		Generalization, percentage of correct responses, mean (SD); range	72.33 (25.69); 30-100	58 (20.07); 30-100	86.67 (22.89); 50-100

^a^OMP: observed mastery probe.

^b^Knowledge, confidence, and usefulness were determined using 4-point scales from the SKIL Survey [36].

### OMP Assessment

#### Acquisition

The Mauchly test indicated that the assumption of sphericity (ie, the equality of variance among difference scores among all testing variables) was not violated (*χ*^2^_2_=0.6; *P*=.74). A Shapiro-Wilk test indicated that the distribution of assessment scores for both groups across the baseline, posttest, and maintenance OMP assessments violated the assumption of normality (*P*≤.02). Nonetheless, we performed a mixed-design ANOVA, given that previous data simulations [53] suggested that ANOVA remains robust when data are not normally distributed. For the baseline and postintervention outcomes for the OMPs, a Levene test indicated that all the measures met the assumption of homogeneity. However, the results of the Levene test suggested that the maintenance scores violated the assumption of homogeneity (*P*=.04). Nonetheless, an ANOVA was performed, given that it is generally robust against violations of homogeneity when sample sizes are equal [54].

We analyzed the data using a mixed-design ANOVA with a between-participants factor of treatment (control and intervention) and within-participants factor of time (baseline, posttest, and maintenance sessions). Large main effects of time (*F*_2,27_=124.154; *P<*.001; η*_p_*^2^=0.816) and treatment (*F*_1,28_=19.281; *P<*.001; η*_p_*^2^=0.408), as well as the interaction effect (*F*_2,28_=8.429; *P<*.001; η*_p_*^2^=0.231) for the OMPs were significant, suggesting a difference in performance between the 2 groups at each time point. Subsequent simple effects tests of within- and between-subjects scores were performed to determine whether the 2 randomly equivalent groups differed.

Within-samples *t* tests revealed a large, significant improvement for the control group between the baseline and posttest sessions (*t*_14_=−10.313; *P*<.001; Cohen *d*=2.66) and a significant decrease in performance between the posttest and maintenance sessions (*t*_14_=2.303; *P*=.02; Cohen *d*=−0.595). Similarly, the intervention group exhibited a large, significant improvement in performance between the baseline and posttest sessions (*t*_14_=−11.859; *P*<.001; Cohen *d*=3.062); however, differences between the posttest and maintenance sessions were not significant (*t*_14_=0.699; *P*=.20), reflecting more stable performance across the 2 probes.

We also performed independent samples *t* tests comparing the performance of the control and intervention groups at each time point. Differences in baseline acquisition OMP were not significant (*t*_28_=0.418; *P*=.40). However, differences between the intervention and control groups were both large and significant at the posttest (*t*_28_=3.653; *P*<.001; Cohen *d*=1.334) and maintenance sessions (*t*_28_=3.807; *P*<.001; Cohen *d*=1.39), suggesting that the VR simulation increased scores relative to the participants who exclusively received the lecture.

#### Generalization

Within-samples *t* tests on the generalization OMP revealed a moderate, significant decrease between the posttest and maintenance probes for the control group (*t*_14_=1.824; *P*=.045; Cohen *d*=−0.471). Changes between the posttest and maintenance sessions for the intervention group were not significant (*t*_14_=0; *P=*.50). Independent samples *t* tests revealed robust, significantly higher scores for the intervention group at both the posttest (*t*_28_=2.488; *P*=.01; Cohen *d*=0.908) and maintenance sessions (*t*_28_=3.647; *P*<.001; Cohen *d*=1.332).

### SKIL Survey

The participants ranked their knowledge, confidence, and understanding of 8 criteria pertaining to the questioning procedure during the baseline and maintenance sessions using the SKIL Survey. We averaged the 8 dimensions of each value across each domain (Table 3). The treatment group reported lower ratings across all scales, relative to the control group, before the intervention. Following the intervention, the ratings across all scales were higher for the treatment group. Statistical comparisons of ratings at baseline and maintenance, performed using the Mann-Whitney *U* test, were initially limited to the confidence domain. The control group exhibited small, significantly higher confidence ratings than the intervention group at baseline, with *U*=64, *P*=.04, and Cohen *r*=0.142. At maintenance, the intervention group exhibited small, significantly higher scores than the control group, *U*=64, *P*=.04, Cohen *r*=0.137. However, inclusion of the knowledge and usefulness scales in the statistical analyses resulted in insignificant adjusted *P* values across all scales, including confidence. Following the Benjamini-Hochberg procedure, we observed no significant differences between knowledge and usefulness either before (*U*=57, *P*=.02 and *U*=83, *P*=.22) or after the intervention (*U*=85, *P*=.25 and *U*=107, *P*=.82).

### Correspondence

Across all conditions and groups, the average correspondence between the acquisition OMP and VMP was 95.98% (SD 7.44%; range 71.43%-100%). The correspondence between generalization OMP and VMP was slightly lower (mean 92.44%, SD 10.30%; range 66.67%-100%). Although not included as measures of performance, we also collected observation data during the probes conducted as part of the intervention (ie, EFPT and DPT). The correspondence between the observed and automated measures during the intervention was high (mean 98.03%, SD 2.96%; range 90.48%-100%).

### Acceptability

Both the treatment (mean 5.73, SD 0.59; range 4-6) and control (mean 5.65, SD 0.82; range 4-6) groups provided high acceptability ratings for the VR portions of their conditions. The treatment (mean 6) and control (mean 5.87, SD 0.35; range 5-6) groups likewise agreed that they had no difficulty using VR.

## Discussion

### Principal Findings

This study compared the effectiveness of a training package featuring VR with didactic instruction as a means of teaching steps in a mathematical questioning strategy. Although participant performance improved following both forms of instruction, the results suggest that gains of the control group deteriorated during maintenance. Differences in performance between the posttest and maintenance sessions favored the VR group, whose scores were significantly higher than those of the participants who received didactic instruction exclusively. A similar pattern of performance was observed for untaught generalization measures. Notwithstanding the results of exploratory analyses featuring all the SKIL Survey scales, the results further suggest that VR contributed to higher confidence in the performance of the procedure. The correspondence between the measures of performance administered by human observers and those administered by AI was generally high. These positive findings, combined with favorable acceptability ratings, support broader applications of VR in education and provide avenues for future inquiry.

Differences observed between the treatment and control groups, although consistent with the positive effects observed in recent literature involving education and VR, were more pronounced in this study than in many previous studies [17,55]. This is likely because of the relatively low responses of participants in the baseline OMP, which mitigated the ceiling effects imposed by the primary measure. In addition, our VR training adapted an evidence-based approach to personnel preparation [29]. Although many simulations are premised on the belief that engagement in a simulated activity with little immediate guidance is beneficial to the learner [56], the findings from this study provide further support for immediate feedback associated with behavioral teaching methods and facilitated through automation [57]. Likewise, the high acceptability of VR across groups corresponds with the growing body of research [50] suggesting that modern VR hardware and approaches to simulation have alleviated motion sickness and other issues associated with earlier VR applications in education [15].

Given that the participants in previous studies required days of exposure to the simulation before mastering the procedure [14], the extent to which the treatment group participants acquired the procedure following a single session was surprising. The findings further suggest that the participants in the control group scored significantly lower on maintenance assessments, whereas scores in the intervention group did not significantly deteriorate. These results must be placed in the context of the limited number of items included in this experiment; nonetheless, the finding that simulation facilitated individualized skill rehearsal—often difficult to arrange in instructor-administered professional development and teacher education programs—provides substantial support for the use of automated opportunities for rehearsal as a supplement to typical instruction. Similar results in the generalization OMP likewise provide qualified support for the VR condition and support the contention that the participants were not merely memorizing appropriate responses based on the original problem. Nonetheless, future work will need to demonstrate the effect of the intervention on a wider range of problems and in practice.

Comparisons between OMPs and VMP revealed acceptable levels of agreement across the conditions. However, the disparities across conditions (ie, higher correspondence in prompting conditions relative to assessments) suggest that the feasibility of AI assessment in professional development, in the absence of extensive classification training, should vary based on the objectives of instruction. That is, the classifier used in this study appears to be suitable for procedures that require minimal deviation from a structured protocol or for determining the extent to which trainees exhibit statements closely aligned with training. As most trainings typically do not extensively assess individuals before instruction (ie, during baseline) and are designed to encourage the exhibition of targeted skills, the capabilities of the current automated system may be appropriate for the typical training context.

### Limitations

This study has several notable limitations. The small convenience sample comprised students from a number of backgrounds that differed considerably from many professionals in education. Therefore, the results may not resemble those likely to be achieved among the targeted population. Given our research questions and the early stage of this scholarship, our emphasis on functionality, rather than external validity, is nonetheless appropriate. In addition, the VR group received more exposure to the procedure than the control group, whose participants did not receive a conventional alternative to the rehearsal provided in VR. However, the more passive training provided to the control group is representative of the instruction that appears in many preservice programs [1] and in-service professional development trainings [2]. The comparison in this study is appropriate because the primary purpose of AI-enhanced VR is to provide opportunities for rehearsal in instructional situations where individualized role-play is not possible. Given the emphasis on speech, we could have implemented the active components of the training using a less immersive platform (eg, a desktop computer). Nonetheless, the current integration of VR and AI contributes to the literature, given (1) the common view that immersion alone provides a benefit to the learner [38] and (2) the limited work regarding the use of AI and VR in teacher training [22]. Additional research is needed to compare immersive simulations with more conventional training approaches and explore the impact of emerging technologies on teacher education and professional development.

### Future Directions

The current VR simulation demonstrates the feasibility of providing instruction in teaching methods using an automated version of an evidence-based training method (ie, BST). Additional work is needed to demonstrate positive effects across a broader range of procedures and settings. The current system analyzes the user based on speech input, which is valuable given the heavy emphasis placed on verbal communication in education. However, the opportunities VR provides to analyze head movement, controller positions, and gaze are what separate the technology from more common platforms. VR training provides opportunities to rehearse behaviors used in practice [58,59] rather than button pressing or other distal representations of authentic performance [13]. Incorporating motion sensing and speech recognition into future work can provide opportunities to train a wide range of complex skills.

Demonstrating the ability of VR-based instruction to promote generalization beyond simulated environments to authentic settings remains a fundamental challenge for the medium [58]. Behavioral theories of learning suggest that prompts and reinforcers can be paired with a variety of antecedents (ie, multiple exemplars) to create antecedent stimulus classes that should nonetheless produce the same response from the learner [39]. This has implications for VR instruction, as learners must (1) be capable of generalizing skills learned in simulations to the actual performance context and (2) apply targeted skills when confronted with situations that do not precisely resemble the situations addressed in training. The ability of VR to alter aspects of a learning simulation across repeated uses (eg, avatar behavior and appearance) has the potential to assist practitioners in generalizing their skills [7]. Randomizing student avatar characteristics (eg, gender and race) may also prevent the bias associated with repeatedly pairing specific types of student behavior with a specific student profile [59]. Addressing such issues will require research that stretches beyond the skills and application contexts featured in this study.

### Conclusions

The findings of this study suggest that an automated, structured approach to VR can improve the acquisition of an educational procedure and participant confidence relative to more conventional, didactic methods. The participants further reported that VR was acceptable and easy to use. Automated assessments of performance generally corresponded to observations conducted by researchers, particularly in conditions where the probes were preceded by guidance regarding appropriate responses. Although promising, additional work is required to explore the effects of AI-enhanced VR on more complex procedures and the cascading effect of such training on practitioners in the field.

## Abbreviations

AI: artificial intelligence
BST: behavioral skills training
DPT: delayed prompting
EFPT: error-free prompting
IOA: interobserver agreement
OMP: observed mastery probe
VMP: virtual mastery probe
VR: virtual reality
